# Methanol Extract of *Commelina* Plant Inhibits Oral Cancer Cell Proliferation

**DOI:** 10.3390/antiox11091813

**Published:** 2022-09-14

**Authors:** Wangta Liu, Yin-Yin Hsu, Jen-Yang Tang, Yuan-Bin Cheng, Ya-Ting Chuang, Jiiang-Huei Jeng, Chia-Hung Yen, Hsueh-Wei Chang

**Affiliations:** 1 Department of Biotechnology, Kaohsiung Medical University, Kaohsiung 80708, Taiwan; 2 Graduate Institute of Medicine, College of Medicine, Kaohsiung Medical University, Kaohsiung 80708, Taiwan; 3 School of Post-Baccalaureate Medicine, Kaohsiung Medical University, Kaohsiung 80708, Taiwan; 4 Department of Radiation Oncology, Kaohsiung Medical University Hospital, Kaohsiung 80708, Taiwan; 5 Department of Marine Biotechnology and Resources, National Sun Yat-sen University, Kaohsiung 80424, Taiwan; 6 School of Dentistry, College of Dental Medicine, Kaohsiung Medical University, Kaohsiung 80708, Taiwan; 7 Department of Dentistry, Kaohsiung Medical University Hospital, Kaohsiung 80708, Taiwan; 8 Department of Dentistry, National Taiwan University Hospital, Taipei 100225, Taiwan; 9 Graduate Institute of Natural Products, Kaohsiung Medical University, Kaohsiung 80708, Taiwan; 10 National Natural Product Libraries and High-Throughput Screening Core Facility, Kaohsiung Medical University, Kaohsiung 80708, Taiwan; 11 Department of Biomedical Science and Environmental Biology, PhD Program in Life Science, College of Life Science, Kaohsiung Medical University, Kaohsiung 80708, Taiwan; 12 Institute of Medical Science and Technology, National Sun Yat-sen University, Kaohsiung 80424, Taiwan; 13 Center for Cancer Research, Kaohsiung Medical University, Kaohsiung 80708, Taiwan; 14 Department of Medical Research, Kaohsiung Medical University Hospital, Kaohsiung 80708, Taiwan

**Keywords:** *Commelina*, oral cancer, apoptosis, DNA damage

## Abstract

Data regarding the effects of crude extract of *Commelina* plants in oral cancer treatment are scarce. This present study aimed to assess the proliferation-modulating effects of the Commelina sp. (MECO) methanol extract on oral cancer cells in culture, Ca9-22, and CAL 27. MECO suppressed viability to a greater extent in oral cancer cells than in normal cells. MECO also induced more annexin V, apoptosis, and caspase signaling for caspases 3/8/9 in oral cancer cells. The preferential antiproliferation and apoptosis were associated with cellular and mitochondrial oxidative stress in oral cancer cells. Moreover, MECO also preferentially induced DNA damage in oral cancer cells by elevating γH2AX and 8-hydroxyl-2′-deoxyguanosine. The oxidative stress scavengers *N*-acetylcysteine or MitoTEMPO reverted these preferential antiproliferation mechanisms. It can be concluded that MECO is a natural product with preferential antiproliferation effects and exhibits an oxidative stress-associated mechanism in oral cancer cells.

## 1. Introduction

Oral cancer is highly prevalent in South-Central Asia, Melanesia, Papua New Guinea, Pakistan, India, and Taiwan [1,2,3]. Oral cancer constitutes a major health problem [4] that increases in both men and women annually and globally [5]. Chemotherapy is a common treatment for oral cancer in addition to surgery and radiotherapy. However, the associated adverse effects of chemotherapy often decrease its therapeutic effectiveness for oral cancer [6]. Identifying anticancer agents with few side effects could help improve oral cancer treatment.

Several natural products are competent for oral cancer treatment with minimal side effects [7]. Some natural products show preferential antiproliferation effects since their anticancer effects are frequently associated with low damage to normal cells. For example, the natural product thymoquinone at 25–300 μM preferentially kills liver cancer but not normal cells [8]. The marine polysaccharide fucoidan at 800–1200 μg/mL inhibits the proliferation of oral cancer cells to a greater extent than normal cells [9]. As indicated, preferential killing agents could improve oral cancer treatment.

The plant genus *Commelina*, belonging to the family Commelinaceae, is a perennial or annual herb. About 170 species have been found in tropical and subtropical areas [10]. This plant has antiseptic, demulcent, emollient, refrigerant, and laxative properties [11]. Phytochemical studies of the genus *Commelina* have isolated alkaloids, terpenoids, steroids, and saponins [12]. However, the anticancer effects of *Commelina* have rarely been investigated.

Recent studies demonstrated that some extracts from *Commelina* could inhibit the proliferation of several cancer cell lines. For example, methanol extract of *C. benghalensis* showed antiproliferation effects against leukemic Jurkat-T cells [13]. Similarly, *n*-hexane and dichloromethane sub-fractions of acetone extracts of *C. benghalensis* at 40 and 120 μg/mL showed an antiproliferation effect against Jurkat-T cells [14]. Ethanol, benzene, *n*-hexane, methanol, and chloroform extracts of *C. benghalensis* root exhibited differential antiproliferation effects in breast and prostate cancer cells [15]. However, most of these studies reported antiproliferation action but lacked the investigation of detailed mechanisms. Moreover, the use of crude extract studies of *C. benghalensis* for oral cancer treatment was rarely reported.

The present study aimed to assess the modulating impacts of Commelina sp. (MECO) methanol extract on oral cancer cell proliferation. The preferential antiproliferation-associated mechanism was investigated using oral cancer and normal cells.

## 2. Materials and Methods

### 2.1. Plant Material, Extraction, and Partition

The aerial parts of *Commelina* sp. were harvested in the Dr. Cecilia Koo Botanic Conservation Center, Pingtung County, Taiwan, in July 2017. A voucher specimen (code no. KMU-NP975 (K051139)) was deposited in the Graduate Institute of Natural Products, College of Pharmacy, Kaohsiung Medical University, Kaohsiung, Taiwan.

The aerial parts of *Commelina* sp. (400 g) were extracted with 1100 mL of methanol at room temperature and then concentrated under reduced pressure. The crude extract (2.6 g) was partitioned between ethyl acetate and water (1:1) to yield an ethyl acetate layer (1.2 g). The ethyl acetate layer was further partitioned between hexanes and 90% MeOH (aq). The 90% MeOH layer was dried by evaporation under reduced pressure using a rotary evaporator (Heidolph, Schwabach, Germany) to obtain MECO (0.3 g). 

### 2.2. HPLC Analysis

HPLC analysis was performed with a Shimadzu Prominence-i LC-2030C instrument equipped (Shimadzu, Kyoto, Japan) with a Sunniest C18 analytical column (5 µm, 4.6 × 250 mm). Water/formic acid (1000:1, *v*/*v*) and methanol/formic acid (1000:1, *v*/*v*) were used as mobile phases A and B, respectively. Linear gradient elution was performed based on the procedure described below: 0–5 min 10% solution B; 5–20 min 10–30% solution B; 20–45 min 30–100% solution B; 45–50 min 100% solution B; 50–60 min 10–100% solution B; flow rate: 1.0 mL/min; detection wavelength: 254 nm; column oven: 40 °C.

### 2.3. Inhibitors for Reactive Oxygen Species (ROS) and Mitochondrial Superoxide (MitoSOX) 

After seeding overnight, cells were pretreated with the ROS inhibitor *N*-acetylcysteine (NAC) (Sigma-Aldrich, St. Louis, MO, USA) [16,17,18,19], a ROS inhibitor, at 10 mM for 1 h and post-treated with MECO for different conditions as shown figure legends. For MitoSOX experiments, cells were seeded overnight, pretreated with MitoTEMPO (Cayman Chemical, Ann Arbor, MI, USA) [17], a MitoSOX inhibitor, at 45 μM for 1 h, and then post-treated with MECO for different conditions as shown in figure legends.

### 2.4. Cell Culture, Cell Viability, and Cell Density Experiments 

Ca9-22 and CAL 27 were used as oral cancer cell lines from ATCC (Manassas, VA, USA) and JCRB Cell Bank (Osaka, Japan). A non-malignant gingival epithelial Smulow–Glickman (S–G) cell line [20,21] was adapted to test the drug safety of anti-oral cancer treatments [22]. The drug safety of MECO was examined by testing S–G cells. Ca9-22 and S–G cells were derived from the gingival area, and CAL 27 cells were derived from the tongue. The culture medium was similar to that of a previous report [23]. CellTiter 96 Aqueous One Solution, an MTS reagent for detecting mitochondrial enzyme activity, was used to estimate cell viability (Promega, Madison, WI, USA) [24,25]. The MTS assay’s seeding cell densities for Ca9-22, CAL 27, and S–G cells were 4, 4, and 6 × 10^3^/well/96-well plate. For flow cytometry, the seeding cell densities for Ca9-22, CAL 27, and S–G cells were 7, 7, and 8 × 10^4^/well/12-well plates. All experiments were incubated with the drug after seeding overnight. The treatment time interval and concentrations are provided, as shown in figure legends.

### 2.5. Cell Cycle Detection

Cellular DNA was stained by 7-aminoactinomycin D (7AAD) (1 μg/mL, 30 min) (Biotium, Inc., Hayward, CA, USA) [26,27] and analyzed by an Accuri C6 flow cytometer (Becton-Dickinson, Mansfield, MA, USA). After cell cycle analysis, subG1 accumulation is used to assess the apoptosis-like status [28].

### 2.6. Apoptosis Detection by Annexin V/7AAD

Annexin V/7AAD kit (Strong Biotech Corporation, Taipei, Taiwan) was a convenient tool for detecting apoptosis [23,29]. Following the manufacturer’s instructions, annexin V-FITC (10 μg/mL) and 7AAD (1 μg/mL) were added to the cell suspension in darkness. Finally, cells were resuspended in 1× PBS for flow cytometry.

### 2.7. Apoptosis Detections by Caspase 3/7 and Caspases 3/8/9

The caspases-Glo^®^ *3/7* peptide-based kit (Promega; Madison, WI, USA) was adopted to determine the activity of activated caspase 3/7 [30]. The activated caspase 3/7 can cleave peptide substrate and generates a light signal for a luminometer (Berthold Technologies GmbH & Co., Bad Wildbad, Germany) detection. Finally, the caspase 3/7 activity was estimated by the cell viability of each treatment.

Additionally, caspases 3/8/9-based flow cytometry kits provided a peptide-based detection for caspases 3/8/9 activations [31]. OncoImmunin kits (Gaithersburg, MD, USA) provided the specific peptide substrates for caspases on 3/8/9. Activated caspases 3/8/9 can cleave substrate to generate fluorescent signals. Finally, cells were resuspended in 1× PBS for flow cytometry.

### 2.8. Oxidative Stress Detection by ROS and MitoSOX

Cells were incubated with DCFH-DA (2 μM) (Sigma-Aldrich; St. Louis, MO, USA) [18,32] and MitoSOX™ Red (5 μM) (Molecular Probes, Invitrogen, Eugene, OR, USA) [23] for 30 min to detect ROS and MitoSOX, respectively. Finally, cells were resuspended in 1× PBS for flow cytometry.

### 2.9. DNA Damage Detection by γH2AX- and 8-Hydroxyl-2′-Deoxyguanosine (8-OHdG)

p-Histone H2AX antibody (Santa Cruz Biotechnology, Santa Cruz, CA, USA) was used to detect γH2AX. Subsequently, fixed cells were incubated with Alexa Fluor^®^ 488-secondary antibody (Cell Signaling Technology, Danvers, MA, USA) [33,34], accompanied by 7AAD (1 μg/mL, 30 min) incubation. 8-OHdG-FITC antibody (1:10,000 dilution) (Santa Cruz Biotechnology) was used to detect 8-OHdG [35]. Finally, cells were resuspended in 1× PBS for flow cytometry.

### 2.10. Statistical Analysis

Significance was determined by ANOVA with Tukey’s HSD post hoc test [35] (JMP12, SAS Institute, Cary, NC, USA). Different lower-case letters reveal significant differences in multi-comparisons.

## 3. Results

### 3.1. HPLC Profile of MECO

**Figure 1:**
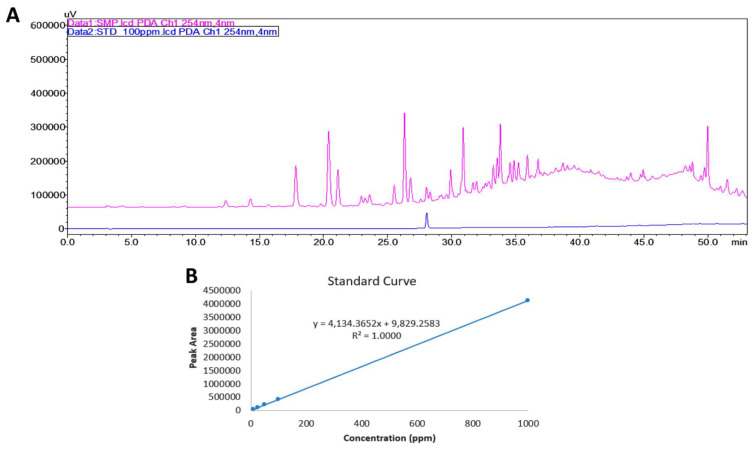
HPLC profile of MECO and active component. (**A**) The HPLC-PDA (254 nm) fingerprint profiles of MECO and indole-3-carboxaldehyde are shown in red and blue, respectively. (**B**) The calibration curve of indole-3-carboxaldehyde.

**Figure 2:**
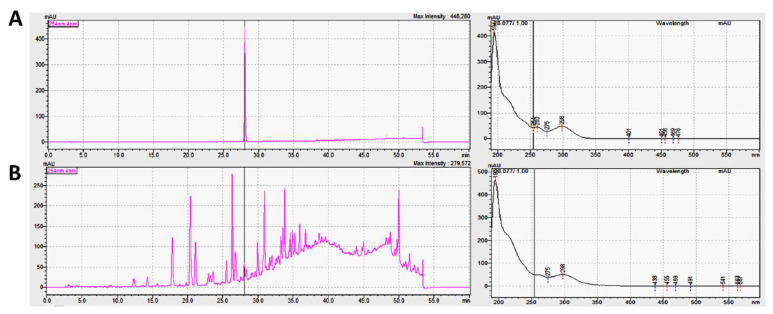
The UV spectra of indole-3-carboxaldehyde and HPLC profile of MECO. (**A**) Standard compound indole-3-carboxaldehyde. (**B**) The fingerprint of MECO.

The HPLC-PDA fingerprint profile of MECO (red line) was performed (Figure 1A). The potential metabolites that may be found in MECO were assessed by data mining using Reaxys (https://www.reaxys.com) (accessed on 1 June 2022) [36]. After searching, indole-3-carboxaldehyde was identified at the top of the result list by searching substances isolated from the genus Commelina and associated with the keyword “cancer”. The HPLC-PDA fingerprint profile of MECO (red line) and in-dole-3-carboxaldehyde (blue line) are shown in Figure 1A. The calibration curve of indole-3-carboxaldehyde is shown in Figure 1B. The retention time for indole-3-carboxaldehyde was 28.03 min. The UV absorption (298 nm and 194 nm) of indole-3-carboxaldehyde showed an identical pattern to those of MECO (Figure 2). The linear equation of the active compounds was y = 4134.37x + 9829.26 (R^2^ = 1). The results showed that indole-3-carboxaldehyde was 9.26 μg/g of MECO.

### 3.2. Proliferation Impact of MECO

**Figure 3:**
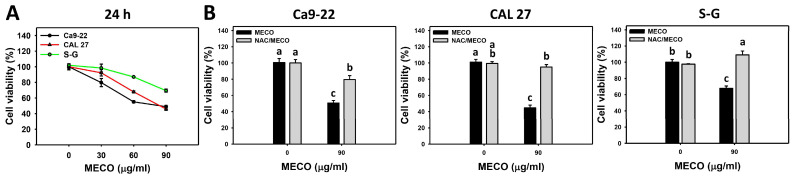
MECO inhibits the proliferation of oral cancer cells. (**A**) MTS assay for MECO. Oral cancer (Ca9-22 and CAL 27) and one non-malignant oral cell (S–G) were tested with control (0.1% DMSO) and MECO for 24 h. (**B**) MTS assay for NAC/MECO. NAC/MECO indicates that cells received the pretreatment of NAC (10 mM, 1 h) before MECO treatment (90 μg/mL) for 24 h. Data = means ± SDs (*n* = 3). Multiple comparison software (JMP12, SAS Institute, Cary, NC, USA) provided lower-case letters for different treatments for assessing significance. Nonoverlapping letters indicate significant results (*p* < 0.05).

The cell viability in the presence of MECO was dose-responsively reduced in oral cancer cells (Ca9-22 and CAL 27). In contrast, MECO showed less viability inhibition in normal cells (S–G) than in oral cancer cells (Figure 3A). Ca9-22 and CAL 27 oral cancer cells possessing high MECO sensitivity were adapted to explore the antiproliferation mechanism in the following experiments. 

The viability between NAC/MECO and MECO was compared to validate the function of ROS in promoting the antiproliferation of MECO-treated oral cancer cells. The ROS inhibitor NAC recovered the MECO-caused antiproliferation (Figure 3B), suggesting that MECO causes ROS-mediated antiproliferation to oral cancer cells.

### 3.3. Cell Cycle Impact of MECO

**Figure 4:**
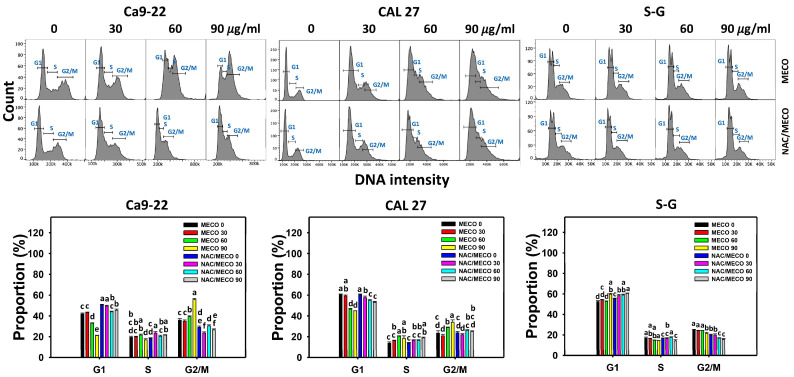
MECO changes cell cycle progression. Oral cancer (Ca9-22 and CAL 27) and non-malignant oral cells (S–G) were tested with control (0.1% DMSO) and MECO for 24 h. NAC/MECO shows cells that received the pretreatment of NAC (10 mM, 1 h) before MECO treatment (90 μg/mL) for 24 h. Data = means ± SDs (*n* = 3). Multiple comparison software provided lower-case letters for different treatments for assessing significance. Nonoverlapping letters indicate significant results (*p* < 0.05).

For Ca9-22 and CAL 27 cells, the G1 phase was decreased, and the G2/M phase was increased at 60 and 90 μg/mL MECO (Figure 4). In contrast, the subG1 population was few and did not measure.

The cell cycle disturbance between NAC/MECO and MECO was compared to validate the function of ROS in regulating the cell cycle progression of MECO-treated oral cancer cells. The ROS inhibitor NAC recovered the MECO-caused G1 decrement for Ca9-22 and CAL 27 cells (Figure 4). Moreover, NAC released the MECO-caused G2/M arrest for Ca9-22 cells.

### 3.4. Annexin V-Apoptosis Impact of MECO

**Figure 5:**
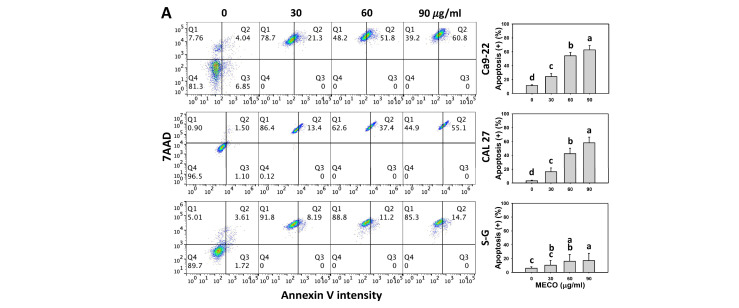

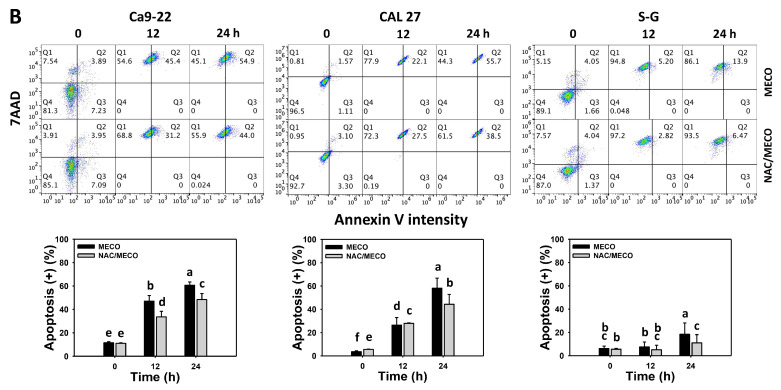
MECO promotes annexin V-detected apoptosis. (**A**) Annexin V assay for MECO. Oral cancer (Ca9-22 and CAL 27) and non-malignant oral cells (S–G) were tested with control (0.1% DMSO) and MECO for 24 h. Apoptosis (%) is the annexin V (+) (%). (**B**) Annexin V assay for NAC/MECO. NAC/MECO indicates that cells received the pretreatment of NAC (10 mM, 1 h) before MECO treatment (90 μg/mL) for 0, 12, and 24 h. Q1, Q2, Q3, and Q4 indicate the necrotic, necrotic/later apoptotic, early apoptotic, and viable cells, respectively [37]. Data = means ± SDs (*n* = 3). Multiple comparison software provided lower-case letters for different treatments for assessing significance. Nonoverlapping letters indicate significant results (*p* < 0.05).

The annexin V-detected apoptosis was increased in MECO-treated oral cancer cells (Ca9-22 and CAL 27), as MECO showed high annexin V (+) compared to normal cells (S–G) (Figure 5A). The annexin V changes between NAC/MECO and MECO were compared to validate the ROS function regulating annexin V-assessed apoptosis of MECO-treated oral cancer cells. The ROS inhibitor NAC recovered the MECO-caused apoptosis (Figure 5B), suggesting that MECO causes ROS-mediated apoptosis in oral cancer cells.

### 3.5. Caspase 3 and Caspase 3/7 Activation Impact of MECO

**Figure 6:**
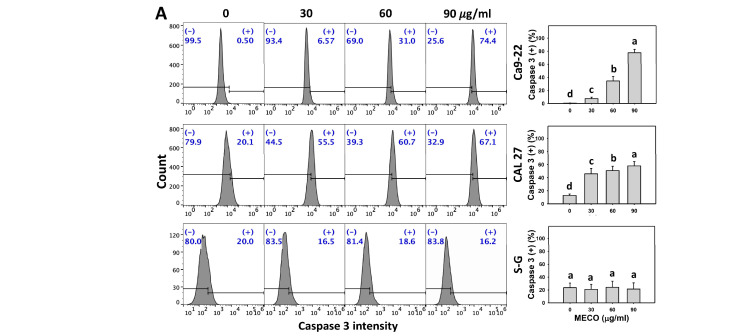

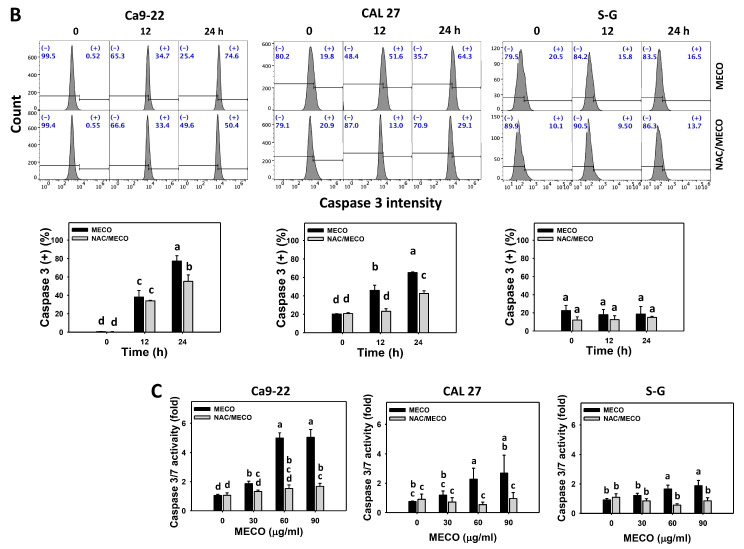
MECO promotes caspase 3 and caspase 3/7 signaling. (**A**) Caspase 3 assays for MECO. Oral cancer (Ca9-22 and CAL 27) and non-malignant oral cells (S–G) were tested with control (0.1% DMSO) and MECO for 24 h. (+) reveals the caspase 3 (+) part. (**B**) Caspase 3 assays for NAC/MECO. NAC/MECO shows cells that received the pretreatment of NAC (10 mM, 1 h) before MECO treatment (90 μg/mL) for 0, 12, and 24 h. (**C**) Caspase 3/7 assays for NAC/MECO. NAC/MECO indicates that cells received the pretreatment of NAC (10 mM, 1 h) before MECO treatment (30, 60, and 90 μg/mL) for 24 h. Data = means ± SDs (*n* = 3). Multiple comparison software provided lower-case letters for different treatments for assessing significance. Nonoverlapping letters indicate significant results (*p* < 0.05).

Using flow cytometry and luminescence analyses, the caspase 3 and caspase 3/7-detected apoptosis was increased in MECO-treated oral cancer cells (Ca9-22 and CAL 27). MECO caused caspase 3 and caspase 3/7 activations in oral cancer cells (Figure 6A,C). Moreover, the MECO induced high caspase 3/7 activation in oral cancer cells compared to normal cells (S–G).

The caspase 3 and caspase 3/7 activity changes between NAC/MECO and MECO were compared to validate the function of ROS in regulating caspase 3 activations of MECO-treated oral cancer cells. The ROS inhibitor NAC recovered the MECO-caused caspase 3 and caspase 3/7 activations (Figure 6B,C), suggesting that MECO causes ROS-mediated caspase 3 activations of oral cancer cells.

### 3.6. Extrinsic and Intrinsic Caspase Activation Impact of MECO

**Figure 7:**
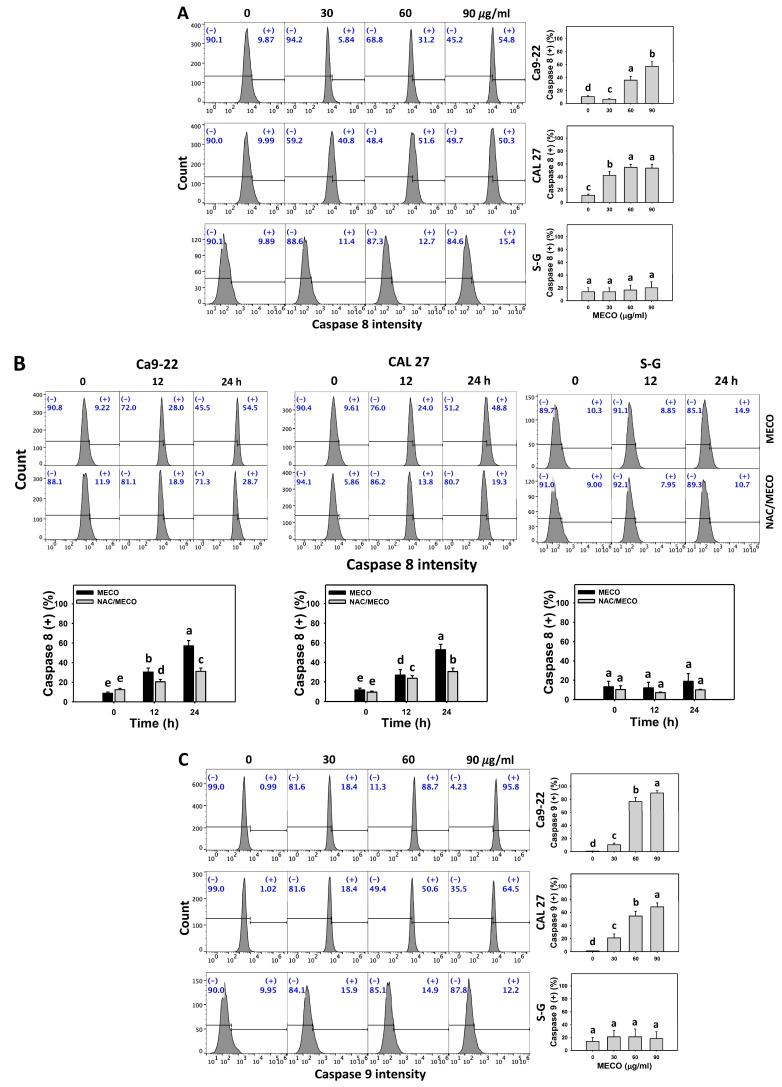

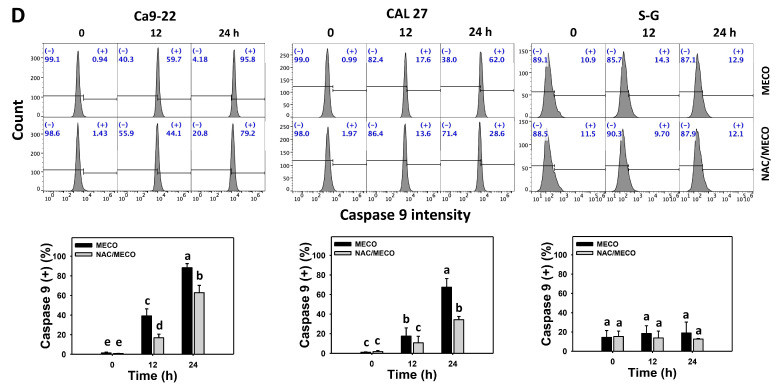
MECO promotes caspases 8/9 signaling. (**A**,**C**) Caspases 8/9 assays for MECO. Oral cancer (Ca9-22 and CAL 27) and non-malignant oral cells (S–G) were tested with control (0.1% DMSO) and MECO for 24 h. (+) reveals the caspases 8/9 (+) part. (**B**,**D**) Caspases 8/9 assays for NAC/MECO. NAC/MECO indicates that cells received the pretreatment of NAC (10 mM, 1 h) before MECO treatment (90 μg/mL) for 0, 12, and 24 h. Data = means ± SDs (*n* = 3). Multiple comparison software provided lower-case letters for different treatments for assessing significance. Nonoverlapping letters indicate significant results (*p* < 0.05).

The caspases 8/9-detected extrinsic and intrinsic apoptosis was increased in MECO-treated oral cancer cells (Ca9-22 and CAL 27) (Figure 7A,C). The caspases 8/9 activity changes between NAC/MECO and MECO were compared to validate the function of ROS in regulating caspases 8/9 activation of MECO-treated oral cancer cells. The ROS inhibitor NAC recovered the MECO-caused caspases 8/9 activation (Figure 7B,D), suggesting that MECO causes ROS-mediated caspases 8/9 activation in oral cancer cells.

### 3.7. ROS Stress Impact of MECO

**Figure 8:**
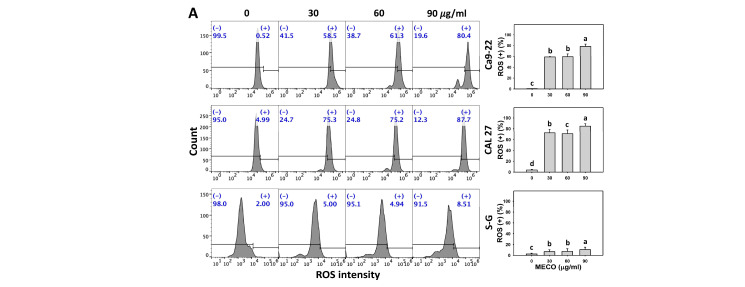

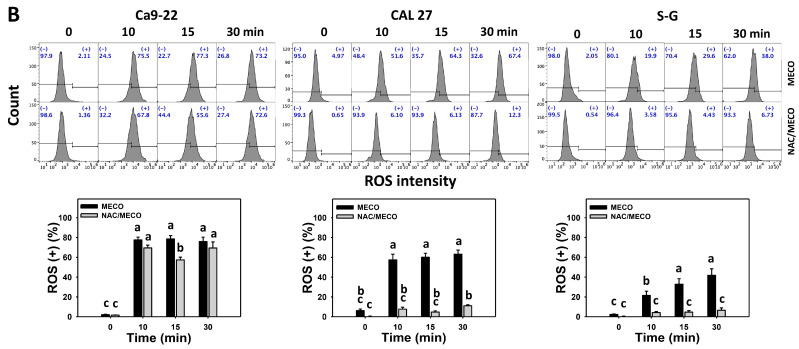
MECO promotes ROS levels. (**A**) ROS assay for MECO. Oral cancer (Ca9-22 and CAL 27) and non-malignant oral cells (S–G) were tested with control (0.1% DMSO) and MECO for 24 h. (+) reveals the ROS (+) part. (**B**) ROS assay for NAC/MECO. NAC/MECO indicates that cells received the pretreatment of NAC (10 mM, 1 h) before MECO treatment (90 μg/mL) for 0, 10, 15, and 30 min. Data = means ± SDs (*n* = 3). Multiple comparison software provided lower-case letters for different treatments for assessing significance. Nonoverlapping letters indicate significant results (*p* < 0.05).

The ROS intensity was increased in MECO-treated oral cancer cells (Ca9-22 and CAL 27), as MECO showed high ROS intensity (+) compared to normal cells (S–G) (Figure 8A). The ROS intensity changes between NAC/MECO and MECO were compared to validate the function of ROS in regulating the ROS intensity of MECO-treated oral cancer cells. The ROS inhibitor NAC reversed the MECO-caused ROS induction (Figure 8B), suggesting that MECO causes ROS generation in oral cancer cells.

### 3.8. MitoSOX Stress Impact of MECO

**Figure 9:**
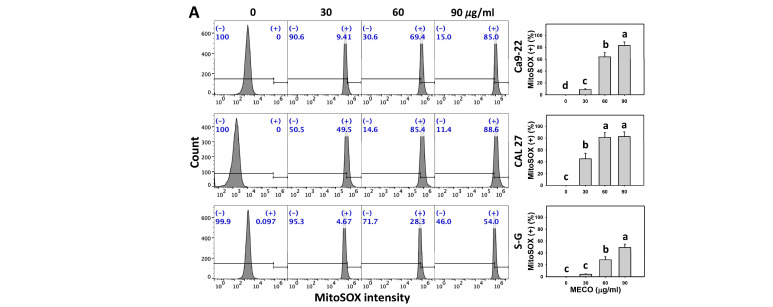

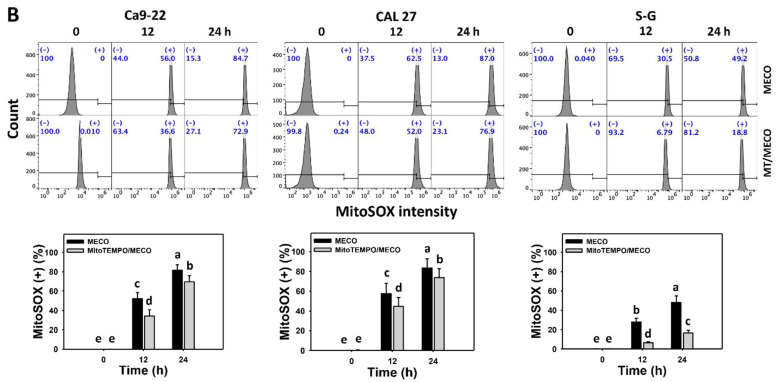
MECO promotes the MitoSOX level. (**A**) MitoSOX assay for MECO. Oral cancer (Ca9-22 and CAL 27) and non-malignant oral cells (S–G) were tested with control (0.1% DMSO) and MECO for 24 h. (+) reveals the MitoSOX (+) part. (**B**) MitoSOX assay for MitoTEMPO/MECO. MitoTEMPO/MECO indicates that cells received the pretreatment of MitoTEMPO (45 μM, 1 h) before MECO treatment (90 μg/mL) for 0, 12, and 24 h. Data = means ± SDs (*n* = 3). Multiple comparison software provided lower-case letters for different treatments for assessing significance. Nonoverlapping letters indicate significant results (*p* < 0.05).

The MitoSOX intensity was increased in MECO-treated oral cancer cells (Ca9-22 and CAL 27), as MECO possessed high MitoSOX intensity (+) compared to normal cells (S–G) (Figure 9A). MitoTEMPO, a MitoSOX inhibitor, is commonly applied to examine the role of MitoSOX in drug response [38]. The MitoSOX intensity changes between MitoTEMPO/MECO and MECO were compared to validate the function of ROS in regulating MitoSOX intensity of MECO-treated oral cancer cells. The MitoSOX inhibitor MitoTEMPO reversed the MECO-caused MitoSOX induction (Figure 9B), suggesting that MECO causes MitoSOX generation in oral cancer cells.

### 3.9. γH2AX Impact of MECO

**Figure 10:**
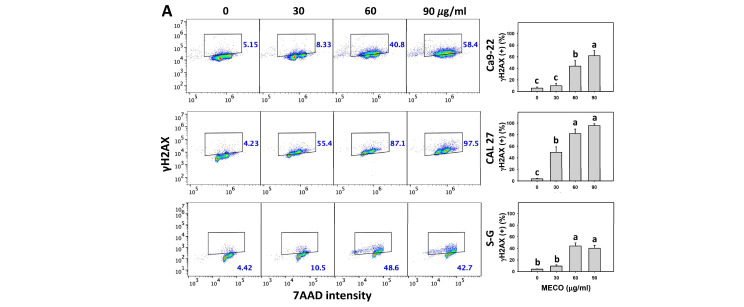

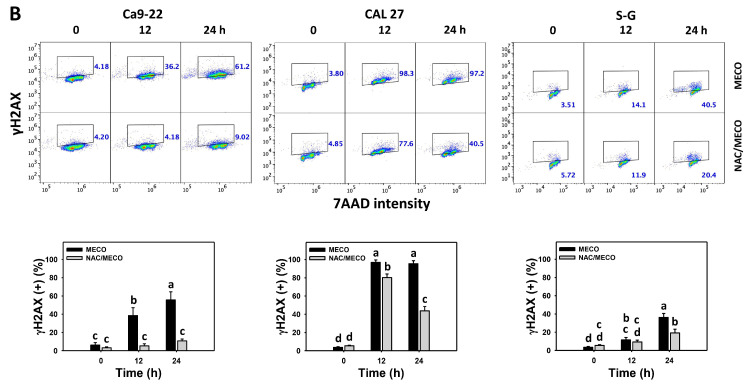
MECO promotes the γH2AX level. (**A**) γH2AX assay for MECO. Oral cancer (Ca9-22 and CAL 27) cells were tested with control (0.1% DMSO) and MECO for 24 h. (+) indicates the γH2AX (+) part. (**B**) γH2AX assay for NAC/MECO. NAC/MECO indicates that cells received the pretreatment of NAC (10 mM, 1 h) before MECO treatment (90 μg/mL) for 0, 12, and 24 h. Data = means ± SDs (*n* = 3). Multiple comparison software provided lower-case letters for different treatments for assessing significance. Nonoverlapping letters indicate significant results (*p* < 0.05).

The γH2AX intensity was increased in MECO-treated oral cancer cells (Ca9-22 and CAL 27) (Figure 10A). The γH2AX intensity changes between NAC/MECO and MECO were compared to validate the function of ROS in regulating γH2AX intensity of MECO-treated oral cancer cells. The ROS inhibitor NAC reversed the MECO-caused γH2AX induction (Figure 10B), suggesting that MECO causes γH2AX generation in oral cancer cells.

### 3.10. 8-OHdG Impact of MECO

**Figure 11:**
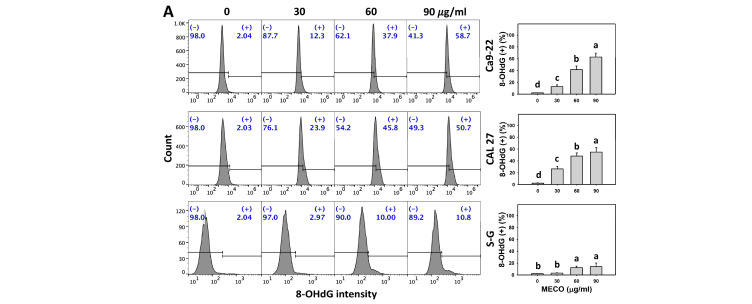

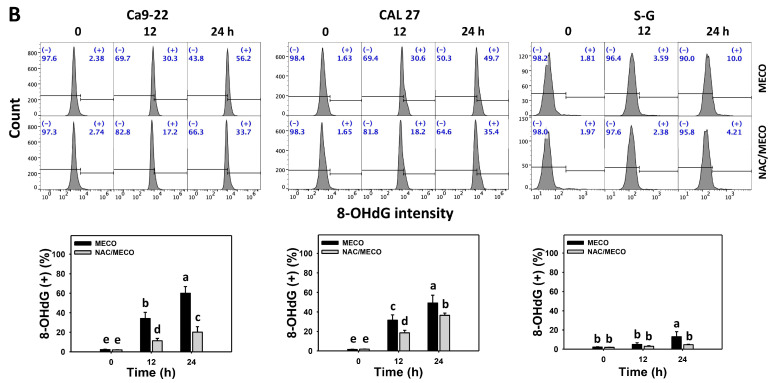
MECO promotes the 8-OHdG level. (**A**) 8-OHdG assay for MECO. Oral cancer cells (Ca9-22 and CAL 27) were tested with control (0.1% DMSO) and MECO for 24 h. (+) indicates the 8-OHdG (+) part. (**B**) 8-OHdG assay for NAC/MECO. NAC/MECO indicates that cells received the pretreatment of NAC (10 mM, 1 h) before MECO treatment (90 μg/mL) for 0, 12, and 24 h. Data = means ± SDs (*n* = 3). Multiple comparison software provided lower-case letters for different treatments for assessing significance. Nonoverlapping letters indicate significant results (*p* < 0.05).

The 8-OHdG intensity was increased in MECO-treated oral cancer cells (Ca9-22 and CAL 27) (Figure 11A). The γH2AX intensity changes between NAC/MECO and MECO were compared to validate ROS’s function in regulating the 8-OHdG intensity of MECO-treated oral cancer cells. The ROS inhibitor NAC reversed the MECO-caused 8-OHdG induction (Figure 11B), suggesting that MECO causes 8-OHdG generation in oral cancer cells.

## 4. Discussion

The anticancer effects of *C. benghalensis* extracts have been rarely investigated, particularly for MECO. Some studies using different *C. benghalensis* extracts were carried out to evaluate anticancer effects (IC_50_ values); however, the detailed molecular mechanisms are rarely addressed [13,14,15]. Notably, the antiproliferation effects of MECO have not been examined in oral cancer cells. The present investigation demonstrated that MECO possessed a preferential antiproliferation effect on oral cancer cells but had a lessened impact on normal cells. Several preferential antiproliferation mechanisms were also investigated. Moreover, the two cancerous cells chosen for the study may have different mechanisms of response to MECO.

### 4.1. MECO Exhibits Oxidative Stress-Modulating Effect

Drug-modulating oxidative stress may perturb redox homeostasis and influence cell viability [39]. A recent review raises the concept that exogenous antioxidant exhibits a dual role in modulating oxidative stress [8]. Exogenous antioxidants from anticancer agents reduce oxidative stress at physiological concentrations but evoke oxidative stress at high cytotoxic concentrations [40]. For example, the grape seed extracts, natural products with potential antioxidant properties, show high cytotoxicity and oxidative stress induction of oral cancer cells at 50 to 400 μg/mL but not the concentration below 10 μg/mL [41]. Hence, natural products with antioxidant properties may have the potential to display anticancer effects. 

Notably, several extracts of *C. benghalensis* were reported to have in vitro antioxidant properties. Ethanol, benzene, *n*-hexane, methanol, and chloroform extracts of *C. benghalensis* root exhibit in vitro antioxidant properties, as shown by their high DPPH scavenging, phenolic, and flavonoid contents [15]. Accordingly, MECO is a *Commelina* sp.-derived methanol extract that may have an antioxidant function. After examination, MECO showed oxidative stress responses to oral cancer cells using several flow cytometry analyses such as ROS and MitoSOX (Figure 8 and Figure 9). Moreover, MECO induces higher oxidative stress in oral cancer cells than in normal cells. These results reveal that MECO is a natural product with the preferential generation of oxidative stress to oral cancer cells. Consequently, this preferential oxidative stress can trigger several responses to the anticancer effects of MECO, which will be discussed later.

### 4.2. MECO Exhibits Antiproliferation-Modulating Effect

Cancer cells generally exhibit a higher level of oxidative stress than normal cells [42,43,44,45,46]. The rise of oxidative stress generated by anticancer agents may exceed cancer cells’ tolerance level but not normal cells [47,48]. Consequently, oxidative stress-modulating agents may show preferential antiproliferation to cancer cells rather than normal cells. Similarly, MECO induced higher oxidative stress in oral cancer cells than in normal cells due to the higher antiproliferation effect in oral cancer cells. It warrants a detailed assessment for testing the future antiproliferative function of MECO in other cancer types.

In leukemic Jurkat-T cells, *n*-hexane and dichloromethane sub-fractions of acetone extracts of *C. benghalensis* showed IC_50_ values of 32.5 μg/mL and 56 μg/mL at 48 h in trypan blue assays, respectively [14]. In breast cancer cells (MDA-MB-231), chloroform, ethanol, and methanol extracts of *C. benghalensis* showed IC_50_ of 134, 180, and 130 μg/mL in 24 h MTT assays [15]. However, these extracts showed no harmful effects on normal breast MCF-10A cells. Similarly, the IC_50_ values of MECO for oral cancer cells (Ca9-22 and CAL 27) and non-malignant cells (S–G) in 24 h MTS assay were 90, 90, and 141.84 μg/mL. They showed a high antiproliferation impact on oral cancer cells compared to normal cells (Figure 1A).

Moreover, the HPLC-PDA fingerprint profile of MECO showed many more compounds in addition to indole-3-carboxaldehyde. The potential for bioactive activities of these compounds cannot be excluded. It warrants a detailed examination of the antiproliferation of these compounds once they are identified. 

Cisplatin is a common chemotherapeutic drug for oral cancer therapy. The drug effectiveness of MECO is evaluated by comparing it to cisplatin. In comparison, cisplatin shows IC_50_ values of 3.55 and 8.58 μg/mL in oral cancer cells (CAL 27 and SCC4), respectively, in a 24 h WST-1 assay [49]. Cisplatin showed IC_50_ values ranging from 4.52 to 60.22 μg/mL for several oral cancer cells (H103, H157, and H314) at 24 h MTT assay [50]. Although MECO is less potent than cisplatin in cancer, the side effects of cisplatin on some cancer patients are concerning, such as cytotoxic effects on the kidney, liver, heart, and other tissues [51]. Alternatively, the combined treatment with a low concentration of cisplatin and other anticancer agents was commonly reported to improve the drug’s effectiveness for cancer treatment. For example, a low dose of cisplatin and nitrated [6,6,6]tricycle derivative (SK2) showed synergistic antiproliferation effects against oral cancer cells [52]. It warrants a detailed evaluation of the anti-oral cancer effects of combined treatment using cisplatin and MECO in the future.

### 4.3. MECO Exhibits Apoptosis and DNA Damage-Modulating Effects 

Oxidative stress is a triggering cause for inducing apoptosis [39,53]. Several kinds of extracts of *C. benghalensis* showed inducible apoptosis function. For example, *n*-hexane and dichloromethane sub-fractions of acetone extracts of *C. benghalensis* trigger apoptosis for leukemic Jurkat-T cells in terms of mRNA expressions for Bax and Bcl-2 genes [14]. However, the detailed mechanisms related to oxidative stress-responsive changes were not investigated.

In addition to oxidative stress, MECO triggered apoptosis, as shown by the results of the present study, including annexin V increment (Figure 5) and caspases 3, 8, and 9 activations (Figure 6 and Figure 7). Consequently, MECO triggered both intrinsic and extrinsic apoptotic signaling in oral cancer cells. Moreover, MECO triggers apoptosis in oral cancer cells, higher than normal cells, suggesting that MECO exhibits a preferential apoptosis-inducible ability in oral cancer cells. However, these findings still need further validation by other methods, such as western blotting for assessing more apoptosis signaling. Using inhibitors of caspases 3, 8, and 9 would also illustrate the contribution of each caspase in MECO-induced apoptosis of oral cancer cells in the future.

Moreover, oxidative stress is also a triggering cause for inducing DNA damage [54]. γH2AX is a sensor for DNA double-strand breaks [55,56], and 8-OHdG is the adduct for oxidative DNA damage [57,58]. In response to drug-induced oxidative stress, γH2AX and 8-OHdG were upregulated in several kinds of cancer cells. For example, fucoidan triggers γH2AX and 8-OHdG-associated DNA damage in oral cancer cells [9]. Similarly, MECO showed similar DNA damage responses to oral cancer cells. MECO triggered more DNA damage in oral cancer cells than normal cells, suggesting that MECO exhibits a preferential DNA damage-inducible ability in oral cancer cells.

S-G cells indeed cause some cell death at high doses (60 and 90 μg/mL) around 87 and 69% cell viability (Figure 1A). According to Figure 5, the cell death of S-G cells may partly attribute to necrosis-like changes. In contrast, the cell death of oral cancer cells (Ca9-22 and CAL 27) may partially attribute to apoptosis-like changes. It warrants a detailed exploration of the roles of apoptosis and necrosis in MECO-induced preferential antiproliferation between oral cancer and non-malignant cells in the future.

### 4.4. MECO Exhibits Cell Cycle-Disturbing Effects

The oral cancer Ca9-22 and CAL 27 cells show a classic G2/M accumulation of cells (Figure 4) that indicates the cells are blocked in mitosis, often characteristic of a cytoskeletal inhibitor. ROS production could also block cell progression through mitosis [59]. It is reasonable that ROS can influence a cell’s progression through the cell cycle by interfering with cycle-dependent proteins such as cyclin-dependent kinases (CDKs) and other cell cycle regulatory proteins [59]. Hence, the ability of NAC to reverse the G2/M block in Ca9-22 and CAL 27 cells (Figure 4).

In the present study, the subG1 is few in MECO-treated oral cancer cells, but the data showed apoptosis in the evidence of annexin V and caspase activations. SubG1 is a convenient indicator for apoptosis. However, the subG1 phenomena are not suitable as the sole indicator for apoptosis [60]. Sometimes, the subG1 population needs more time to accumulate. For example, the subG1 population is absent for 24 and 48 h but dramatically accumulates at 72 h for (−)-anonaine-treated lung cancer H1299 cells [61].

### 4.5. Differential Oxidative Stress Controls Differential Antiproliferation Mechanisms

In the present investigation, MECO induced several oxidative stress responses in oral cancer cells, such as antiproliferation, altered cell cycle, oxidative stress, apoptosis, and DNA damage. The dependence of oxidative stress in these responses was validated by NAC or MitoTEMPO pretreatment because NAC or MitoTEMPO reverted all these MECO-induced changes to some extent. Consequently, MECO partly causes differential antiproliferation in oral cancer cells by inducing oxidative stress. MECO also caused ROS-mediated cell cycle disturbance to oral cancer cells.

Reversal of the inhibitory effects of MECO was variable by the ROS inhibitor NAC, about 50% for cell proliferation but much less for annexin V binding. For the caspase activations, it was about 30 to 50%. For ROS intensity, it was very low for Ca9-22 cells and almost a complete loss of the MECO response for CAL 27 cells. However, both cell types showed significant ROS production increases in the presence of MECO (Figure 8). Since NAC is a cysteine and glutathione (GSH) precursor [62], NAC-converted GSH can alleviate ROS levels depending on the degree of GSH conversion. The NAC to GSH conversion probably had different rates between Ca9-22 and CAL 27 cells. Consequently, the ROS decrement by NAC was higher in CAL 27 cells than in Ca9-22 cells. It warrants a detailed assessment of the GSH levels between the two cancer cell lines following MECO and NAC/MECO treatments in the future. 

## 5. Conclusions

In the present study, we firstly demonstrated that MECO modulated the proliferation of oral cancer cells by causing higher oxidative stress, apoptosis, and DNA damage than in normal cells. These preferential changes were confirmed to be oxidative stress-dependent. Therefore, through oxidative stress-associated responses, MECO is an effective antiproliferation agent for oral cancer cells.

## Data Availability

Data are contained within the article.
